# Altered microRNA and target gene expression related to Tetralogy of Fallot

**DOI:** 10.1038/s41598-019-55570-4

**Published:** 2019-12-13

**Authors:** Marcel Grunert, Sandra Appelt, Ilona Dunkel, Felix Berger, Silke R. Sperling

**Affiliations:** 1 Cardiovascular Genetics, Charité - Universitätsmedizin Berlin, Berlin, Germany; 2 Berlin Institute of Health (BIH), Berlin, Germany; 3 DZHK (German Centre for Cardiovascular Research), partner site Berlin, Berlin, Germany; 4 Cardiovascular Genetics, Department of Vertebrate Genomics, Max Planck Institute for Molecular Genetics, Berlin, Germany; 5 Department of Pediatric Cardiology, German Heart Institute Berlin and Department of Pediatric Cardiology, Charité - Universitätsmedizin Berlin, Berlin, Germany; 6 Department of Biology, Chemistry, and Pharmacy, Freie Universität Berlin, Berlin, Germany

**Keywords:** Gene expression, Gene regulation

## Abstract

MicroRNAs (miRNAs) play an important role in guiding development and maintaining function of the human heart. Dysregulation of miRNAs has been linked to various congenital heart diseases including Tetralogy of Fallot (TOF), which represents the most common cyanotic heart malformation in humans. Several studies have identified dysregulated miRNAs in right ventricular (RV) tissues of TOF patients. In this study, we profiled genome-wide the whole transcriptome and analyzed the relationship of miRNAs and mRNAs of RV tissues of a homogeneous group of 22 non-syndromic TOF patients. Observed profiles were compared to profiles obtained from right and left ventricular tissue of normal hearts. To reduce the commonly observed large list of predicted target genes of dysregulated miRNAs, we applied a stringent target prediction pipeline integrating probabilities for miRNA-mRNA interaction. The final list of disease-related miRNA-mRNA pairs comprises novel as well as known miRNAs including miR-1 and miR-133, which are essential to cardiac development and function by regulating *KCNJ2*, *FBN2*, *SLC38A3* and *TNNI1*. Overall, our study provides additional insights into post-transcriptional gene regulation of malformed hearts of TOF patients.

## Introduction

Over the last century, cardiovascular diseases have slowly overtaken infectious diseases as the leading cause of death worldwide. Among various subdivisions, congenital heart diseases (CHDs) feature prominently and contribute appreciably to the mortality rates^1^. The symptoms vary from life-threatening at one extreme to somewhat benign. One severe malformation is Tetralogy of Fallot (TOF). This heart defect is the most common cyanotic one with a prevalence of ~0.3 per 1.000 live births, accounting for 7–10% of all CHDs^2,3^. TOF is characterized by four structural abnormalities, namely a narrowing of the right outflow tract (pulmonary stenosis), a ventricular septal defect (VSD), a displacement of the aorta to the right side over the VSD (an overriding aorta) and a hypertrophy of the right ventricle^3,4^. Like the majority of CHDs, TOF is most probably caused by an interplay of multiple subtle genetic, structural genomic or epigenetic alterations with environmental stimuli^5–9^. Moreover, etiologies of CHDs also include the disruption of developmentally transcriptional regulation, which requires precise spatiotemporal control of gene expression^8^. Here, post-transcriptional regulation by microRNAs (miRNAs) has emerged as an important regulator in development and maintaining function of the human heart^8,10^.

MicroRNAs are small, non-coding RNAs that non-precisely complementary bind to the 3′ untranslated regions of target mRNA, which results in translational repression or mRNA degradation^11^. Studies have been performed on tissue-specific and circulating miRNAs as novel diagnostic and prognostic biomarkers^12–14^. Recently, altered expression levels of circulating miRNAs were found in blood of TOF patients after surgical repair being mainly surgical related. These miRNAs differ compared to those seen in the right ventricle (RV) of the patients^15^ and show limited correlation with cardiac functional indices assessed by echocardiography^16^. Studies of cardiac tissue of TOF range from single candidate approaches to microarray studies^17–21^ up to whole small RNA transcriptome analysis using high-throughput sequencing^22^. Overall, these studies show a broad range of altered miRNAs in TOF with heterogeneous results between them.

Here, we profiled genome-wide the whole transcriptome in cardiac tissues. We analyzed the relationship of miRNAs and mRNAs in RV tissue of a homogeneous group of non-syndromic TOF patients compared to normal right and left heart controls. To narrow down the large list of putative functional target genes of differentially expressed miRNAs, the expression is correlated to mRNA expression profiles of cardiac tissue from the same group of patients and controls. By this, we add a novel layer of data as this comparison was so far limited.

## Results

### Quality, mapping and annotation of sequencing data

**Figure 1:**
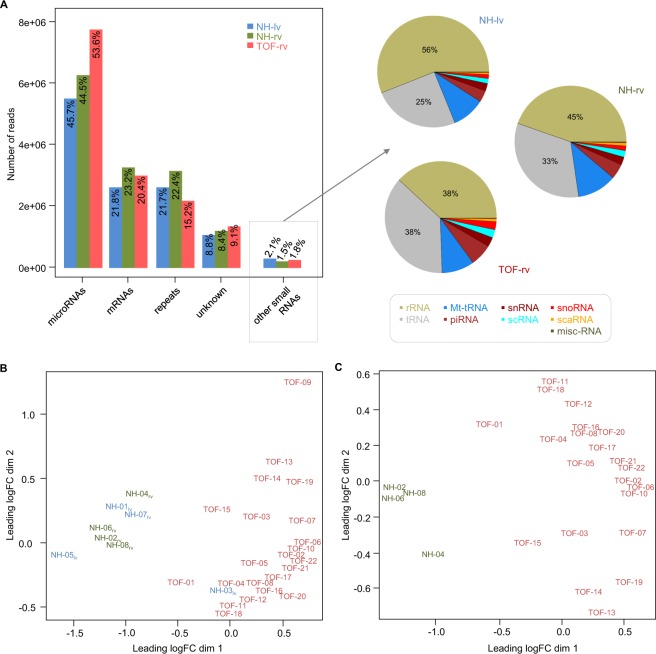
Annotation of mapped reads and multi-dimensional scaling of normalized read counts. (**A**) Overview of annotated mapped read sequences of miRNAs, mRNAs, repeats, unknown origin and other small RNAs in left ventricle (NH-lv) as well as right ventricle of normal hearts (NH-rv) and TOF patients (TOF-rv). Multi-dimensional scaling before (**B**) and after removal of outliers (NH-03 and TOF-09) as well as NH-lv samples in general. (**C**) Scaling based on TCC normalized miRNA read counts. LogFC: log fold change; lv: left ventricle; NH: normal heart; rv: right ventricle; TOF: Tetralogy of Fallot.

Small RNA sequencing of RV tissue of 22 isolated TOF patients (TOF-rv) as well as tissue from RV and LV of four normal hearts (NH-rv and NH-lv, respectively) serving as controls (Supplementary Fig. S1) revealed on average 15 million reads per sample. Sequencing data suggest a good sequencing quality for all samples, with on average 4.1 million unique read sequences per samples (Table 1). On average, 12.8 million (86%) of the input reads could be mapped to human reference genome (Table 1). The read length distribution of mapped reads is representative for small RNA sequences, meaning 18–36 nucleotides (nt) (Supplementary Fig. S2A). Using annotations from miRBase, the majority of the mapped reads could be assigned to known mature miRNA sequences (Fig. 1A left), comprising a read length distribution between 18 and 25 nt with a peak near the average mapped read length of 22 nt (75.2% of the mapped reads), which is representative for mature sequences (Supplementary Fig. S2B). After annotation of miRNAs, the remaining mapped reads were assigned to other known non-coding RNAs, mRNA sequences and genomic repeats. The most abundant classes of non-coding RNAs except miRNAs are rRNAs and tRNAs (Fig. 1A right). In general, the amount of non-coding RNAs except miRNAs is small (~1.8%), which indicates an accurate library preparation and low contamination over all small RNA-seq libraries. The relatively high number of reads assigned to mRNAs and genomic repeats could be attributed to ambiguously mapped reads, unannotated microRNAs or short mRNA degradation products.

**Table 1:** Small RNA sequencing read statistics and mapping to human reference genome (hg19).

Sample	Tissue	Reads	Unique read sequences	GC (in %)	Sequence duplication levels (in %)	Mapped reads	
NH-01	LV	14,111,358	2,865,658	30	70	12,891,425	91%
NH-03	LV	12,230,279	3,343,404	43	47	9,058,367	74%
NH-05	LV	13,936,063	3,260,509	31	60	10,387,224	75%
NH-07	LV	14,794,093	3,805,985	31	63	12,490,569	84%
*mean in NH-lv*		*13,767,948*	*3,318,889*	34	60	*11,206,896*	*81%*
NH-02	RV	16,270,049	5,396,081	30	55	14,358,788	88%
NH-04	RV	12,940,172	2,303,102	31	75	11,688,828	90%
NH-06	RV	14,475,968	3,911,569	29	65	13,181,979	91%
NH-08	RV	14,890,970	3,720,101	30	67	13,637,109	92%
*mean in NH-rv*		*14,644,290*	*3,832,713*	30	66	*13,216,676*	*90%*
TOF-01	RV	15,618,489	6,185,939	35	49	14,155,706	91%
TOF-02	RV	14,247,548	3,616,342	43	65	11,871,100	83%
TOF-03	RV	16,154,319	5,027,091	35	55	13,947,241	86%
TOF-04	RV	13,530,942	3,037,292	39	67	12,062,813	89%
TOF-05	RV	13,178,983	2,458,431	39	75	11,943,094	91%
TOF-06	RV	15,681,483	4,165,561	43	59	13,239,917	84%
TOF-07	RV	14,459,386	2,717,782	40	67	12,322,079	85%
TOF-08	RV	14,893,149	4,646,465	40	59	12,967,004	87%
TOF-09	RV	16,226,821	6,988,240	38	33	12,957,736	80%
TOF-10	RV	15,467,857	3,978,431	43	58	12,947,678	84%
TOF-11	RV	14,989,342	5,846,310	44	56	12,597,864	84%
TOF-12	RV	14,684,351	5,124,711	44	58	12,110,460	82%
TOF-13	RV	15,412,115	3,826,079	35	60	13,811,681	90%
TOF-14	RV	14,722,727	3,724,013	35	56	12,538,399	85%
TOF-15	RV	14,982,308	3,698,674	34	57	13,423,968	90%
TOF-16	RV	16,914,098	4,066,034	41	58	13,911,659	82%
TOF-17	RV	15,860,118	3,046,123	42	68	13,550,609	85%
TOF-18	RV	16,542,142	5,849,400	40	50	14,442,540	87%
TOF-19	RV	14,560,854	3,479,567	41	61	12,096,704	83%
TOF-20	RV	17,891,078	5,110,475	42	55	14,372,964	80%
TOF-21	RV	14,033,794	2,975,214	41	68	12,290,647	88%
TOF-22	RV	16,296,019	3,863,386	42	63	13,766,977	84%
*mean in TOF-rv*		*15,288,542*	*4,246,889*	40	59	*13,060,402*	*86%*

LV: left ventricle; NH: normal heart; RV: right ventricle; TOF: Tetralogy of Fallot.

### Quantification of miRNAs

Overall, a higher proportion of reads associated to miRNAs was found in TOF patients compared to controls (Fig. 1A). In total, 657 expressed miRNAs (Tag Count Comparison [TCC] > 1) were found in TOF-rv or normal heart controls. After miRNA quantification and read count normalization, the samples were assessed based on multidimensional scaling (Fig. 1B,C). We identified the samples NH-03 (left ventricle) and TOF-09 (right ventricle) as clearly separated from the others in the first and second dimension (Fig. 1B), respectively, which may be related to the sequencing results. (Table 1). Thus, both samples were classified as outliers and were subsequently removed from further analysis.

### Differential expression analysis

**Figure 2:**
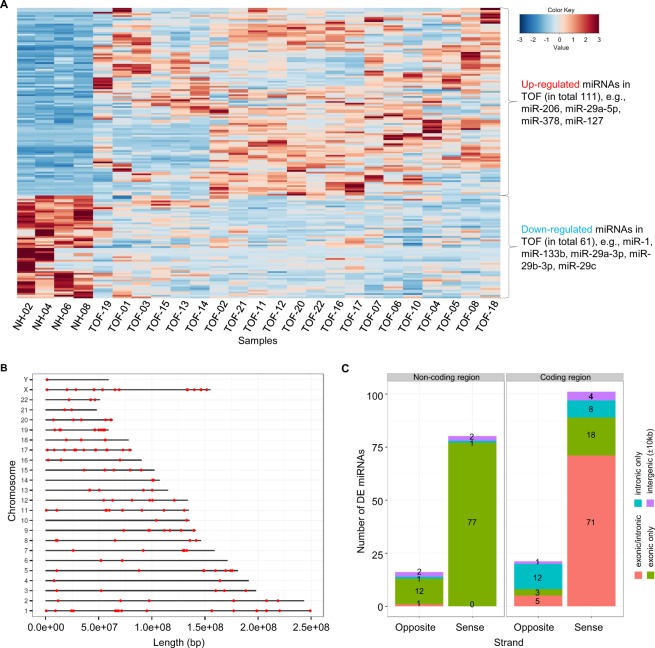
Significantly differentially expressed miRNAs in TOF-rv compared to NH-rv. (**A**) Heatmap based on 172 differentially expressed miRNAs in right ventricular tissue of TOF patients and normal heart controls. All read counts are based on TCC normalized expression values. The heatmap was produced using McQuitty’s similarity cluster analysis with a correlation metric distance. The data were dual scaled to the limits -3,3 before clustering. (**B**) Genomic locations of miRNAs over all human chromosomes. (**C**) Locations of miRNAs from where they are transcribed, distinguishing between coding and non-coding regions; intronic, exonic or intergenic regions; and strand position.

To define differential expression between TOF patients and controls, 21 TOF samples and four normal heart samples from the right ventricle were compared. The analysis revealed 172 significantly differentially expressed miRNAs (adjusted p-value < 0.05 and fold change ≥ 1.5; Supplementary Table S1). Most of these miRNAs were up-regulated in TOF (in total 111) including several heart- and muscle-relevant miRNAs (e.g., miR-206, miR-29a-5p, miR-378, and miR-127). Approximately one third of the miRNAs were down-regulated in TOF (in total 61) including important miRNAs such as miR-1, miR-133b, miR-19a/b-3p, and miR-29c (Fig. 2A). We further checked possible expression differences of identified miRNAs between patients with and without dilated right ventricle or muscular intra-ventricular septum defect. However, we found overall no obvious or significant expression difference based on hierarchical clustering as well as mean expression correlations (Supplementary Fig. S3). This holds true for other clinical parameters, including for example O_2_ saturation, systolic pressure in RV and pulmonary valve morphology, where one can identify sub-classes based on phenotype data (Supplementary Fig. S1). In general, the differentially expressed miRNAs are randomly distributed over all chromosomes of the human genome and no clusters could be identified (Fig. 2B). Moreover, the majority (in total 122) overlap with protein-coding genes (Fig. 2C). Most of the miRNAs are located on the same strand of both protein-coding or non-protein-coding host genes. However, miRNAs located on non-protein coding genes were nearly exclusively located in exonic regions of gene transcripts whereas miRNAs on protein-coding genes were mostly located in exonic/intronic regions (Fig. 2C).

For the 172 significantly differentially expressed miRNAs, we searched for possible age-related candidates with a continuous increase or decrease of the normalized reads counts from infant TOF hearts to young and mid-age adult normal hearts (i.e., TOFs 0–3 y < NH-04 18 y < NH-06 20 y < NH-02 25 y < NH-08 37 y). Among the 111 up-regulated miRNAs in TOF, we only found three miRNAs (miR-3124-5p, miR-3127-5p and miR-618), which harbor decreased reads counts from infant TOF hearts to the mid-age adult heart of NH-08. For the 61 down-regulated miRNAs, there are five miRNAs (let-7a-3p, miR-126-3p, miR-140-5p, miR-21-5p and miR-98-5p) with continuous increased read counts from infant TOF hearts to the mid-age adult normal heart. Whether differential expression of these miRNAs between TOF and normal heart (only 5 out of 172) is age- or disease-related cannot be clearly determined and therefore, we did not exclude these miRNAs from further analysis.

**Figure 3:**
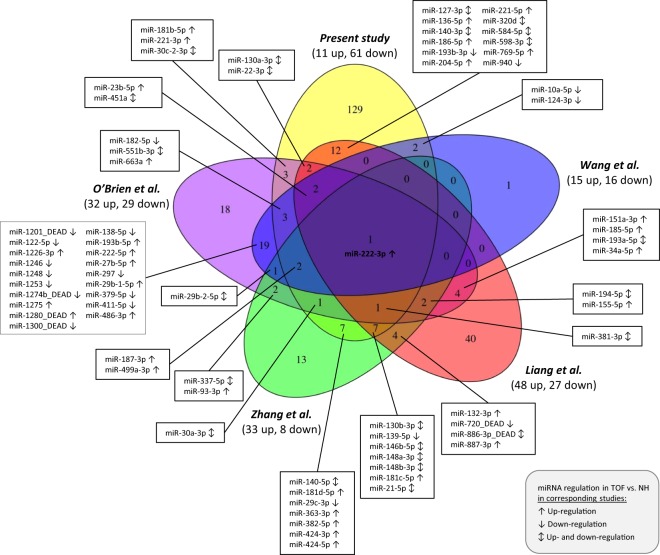
Overlap of significantly differentially expressed miRNAs in the right ventricle of TOF patients versus normal hearts of four studies.

Differential expression analysis between right and left ventricles of normal hearts revealed exclusively miR-223-3p being down-regulated in NH-rv compared to NH-lv (mean TCC read count of 64 in NH-rv and mean TCC read count of 329 in NH-lv; adjusted p-value of 0.025). Interestingly, miR-223-3p is also down-regulated in the TOF-rv compared to NH-rv, which is characterized by a hypertrophic cardiac RV mass and increased ventricular pressure that is in line with the comparison of NH-rv and NH-lv. This miRNA regulates the Glucose Transporter 4 (Glut4) protein expression and cardiomyocyte glucose metabolism^23^. Approximately 25% of the differentially expressed miRNAs (43 out of 172) overlaps with results from other studies based on right ventricular tissue of TOF patients versus normal hearts, with only one miRNA altered in all studies, namely miR-222-3p (Fig. 3)^17,18,20,24^. Additionally, we compared differential expressed miRNAs to altered circulating miRNAs in maternal serum of pregnant women with fetal CHDs including TOF^25,26^. This revealed only two common TOF specific expressed miRNAs, namely miR-22 and miR-29c. They are down-regulated in heart tissue of our study while up-regulated in maternal serum in the study by Zhu *et al*.^26^.

### Target prediction of differentially expressed miRNAs

**Figure 4:**
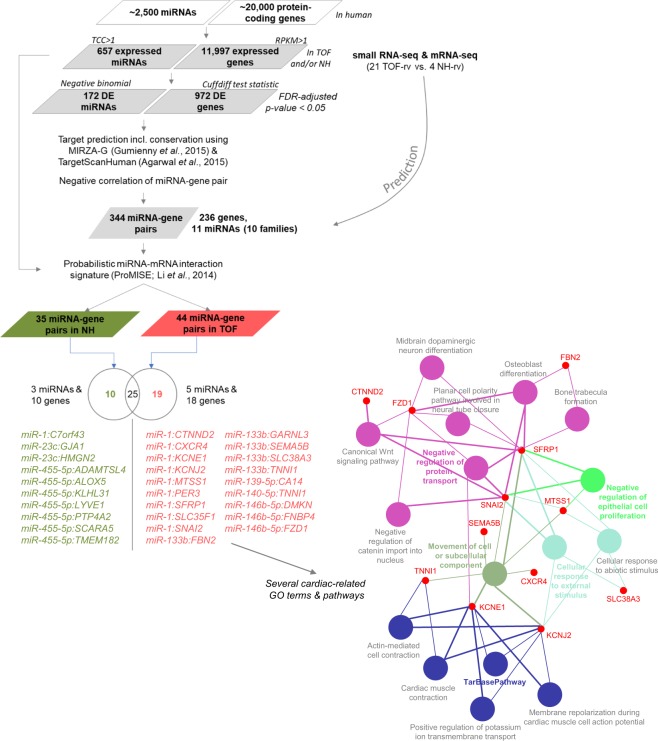
Target prediction workflow for significantly differentially expressed miRNAs (n = 172) and mRNAs (n = 972) in the right ventricle of TOF patients compared to normal heart controls.

The set of 172 significantly differentially expressed miRNAs between TOF-rv and NH-rv was further selected for target prediction using MIRZA-G^27^ and TargetScanHuman^28^ and linking to 972 mRNAs, which are also significantly differentially expressed in the right ventricle of these patients and controls^5,7^. Note that the already published mRNA-seq data^5,7^ were generated from cardiac tissue of the same TOF and normal hearts. Following a stringent approach, we only selected negatively correlated miRNA-mRNA pairs (i.e., up-regulated miRNA and down-regulated mRNA, or *vice versa*), which corresponds to the main regulatory mechanism of miRNAs^29^. This prediction and filtering approach resulted in 344 pairs comprising 235 genes (protein-coding mRNAs) and only 11 miRNAs from 10 families (Fig. 4). The list of miRNA-gene pairs includes already validated pairs such as miR-1 & *GJA1* (Gap Junction Protein Alpha 1), miR-140-5p & *KLF9* (Kruppel Like Factor 9) and miR-1 & *KCNJ2* (Potassium Voltage-Gated Channel Subfamily J Member 2), which are also included in public databases such as DIANA/miRTarBase^30^. *GJA1* and *KCNJ2*, both targeted by miR-1, are two ion channel genes, which are known to play a major role in cardiac disease and development^31^.

Considering that mRNAs are typically targeted by many miRNAs and each miRNA targets multiple mRNAs, identified miRNA-gene pairs are part of co-regulation and interaction networks including competition between mRNAs and miRNAs. Thus, the target prediction workflow was extended to include a probabilistic model to identify miRNA-mRNA interaction signatures (ProMISe)^32^. The results are miRNA-gene pairs, which are most likely to occur given the whole expressed miRNA (n = 657) and mRNA (n = 11,997) background. Here, cases that are uniquely predicted in either TOF patients or normal heart controls are most interesting. This holds true for 19 pairs in the TOF patients (comprising 5 miRNAs and 18 genes) and 10 pairs in the controls (comprising 3 miRNAs and 10 genes) (Fig. 4). The final 18 target genes in the TOF patients are significantly enriched for several cardiac-related Gene Ontology (GO) terms and pathways (Fig. 4).

### Novel miRNAs in the human heart

**Figure 5:**
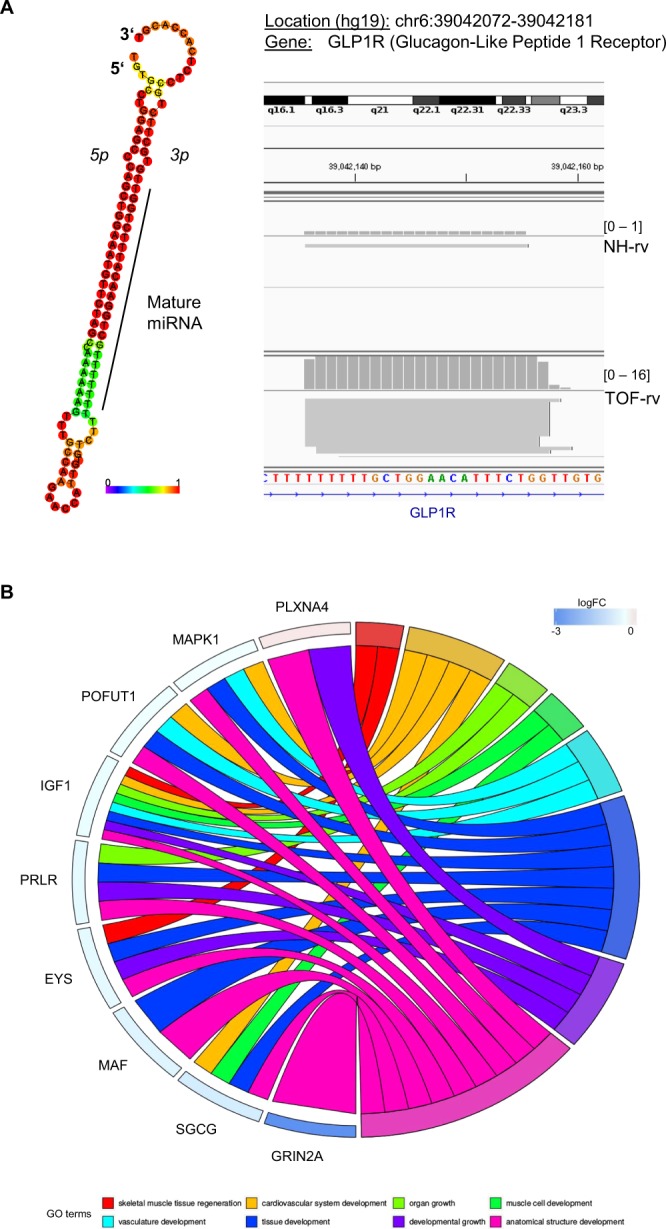
Predicted novel miRNA candidate located at chromosome 6 and in an intron of GLP1R. (**A**) The centroid secondary structure drawing encoding base-pair probabilities based on RNAfold WebServer is given on the left. A graphical representation of the novel miRNA location with read counts over all NH-rv and TOF-rv samples is given on the right. (**B**) Interaction-like graph based on GOplot for processes and associated target genes of the novel miRNA filtered for significantly enriched cardiac- and development-related GO terms.

Approximately 8–9% of the mapped small RNA-seq reads could not be assigned to known miRNAs, other small non-coding RNAs, mRNAs or genomic repeats (Fig. 1A). Thus, we searched for novel miRNAs over all heart samples using a fold- and scoring-based approach^33^, which revealed three novel miRNA candidates. Moreover, all three miRNAs are significantly differentially expressed in the right ventricle of TOF patients compared to normal heart controls (Fig. 5 and Supplementary Figs. S4–7). Most interestingly, the target prediction for the novel miRNA candidate located on chromosome 6 and in an intron of *GLP1R* (Glucagon-Like Peptide 1 Receptor) (Fig. 5A; Supplementary Fig. S5) revealed 15 target genes (Supplementary Table S2), which are enriched for several cardiac-related significantly enriched GO terms (e.g., cardiovascular system development) and pathways (e.g., cardiac hypertrophic response, hypertrophic and dilated cardiomyopathy, miRNAs in cardiomyocyte hypertrophy). Moreover, the interaction-like graph of processes and associated genes filtered for cardiac and developmental related GO terms shows that the target genes belong to the anatomical structure development and developmental growth (Fig. 5B). The target genes (in total 10; Supplementary Table S2) of the novel miRNA candidate located on chromosome 16 and overlapping the long intergenic non-protein coding RNA 922 (*LINC00922*) (Supplementary Figs. S4A and S6) are significantly enriched for pathways such as ‘assembly of collagen fibrils and other multimeric structures’ and ‘collagen formation’. Significantly enriched GO terms for this miRNA are ‘sarcoplasm’ and ‘contractile fiber part’ among others. For the novel miRNA candidate located on chromosome 1 and in an intron of *NKAIN1* (Na+/K+ Transporting ATPase Interacting 1) (Supplementary Figs. S4B and S7), no significantly enriched GO terms and only ‘translation factors’ for the pathways were found based on the 31 target genes (Supplementary Table S2).

## Discussion

Post-translational regulation of gene expression by non-coding RNAs plays an important role in multiple cellular pathways and diseases. Here, genome-wide small RNA sequencing of cardiac biopsies from isolated TOF patients and healthy unaffected individuals was performed to investigate the role of miRNAs in the normal and malformed human heart. Deep sequencing revealed mostly up-regulated miRNAs in TOF compared to normal heart controls, which is in line with other heart failure studies^34^. Furthermore, our study design enabled us to find several miRNAs that are well-known for non-congenital heart diseases^13,34,35^ to be also altered in TOF patients.

Recently, sexual differences in miRNA expression of TOF hearts was shown, with miR-1 and miR-133 accounting for the most variance between sexes^22^. In general, we do not observe significant sex-specific differences of miRNA expression profiles in our TOF cohort. For miR-1, the fold change of mean normalized read counts between male and female TOF hearts is 1.16, although there is a difference of approximately 800k reads (5.5 million normalized read counts for TOFs with 5.9 million reads for male TOFs and 5.1 million reads for female TOFs compared to 8.9 million read counts for NHs with 9.7 million reads for male NHs and 8.0 million reads for female hearts). For miR-133b, the fold change is 1.95 but there is only a difference of 471 reads in total (741 normalized read counts for TOFs with 966 reads for male TOFs and 495 reads for female TOFs compared to 2,107 read counts for NHs with 2,427 reads for male NHs and 1,787 reads for female hearts). However, both miRNAs are significantly down-regulated in TOF versus normal heart, which also holds true for a gender-specific analysis. In comparison to the normal heart, which is the subject of this study, sex-specific differences of miRNA expression profiles can be ignored as a balanced number of male and female TOF hearts (11 male and 10 female TOFs) and normal hearts (2 male and 2 female NHs) is compared.

Among the significantly altered miRNAs in TOF hearts are the down-regulated miR-1 & miR-133b (both associated with cardiac hypertrophy)^31^ and miR-29a (involved in the control of cardiac fibrosis)^31^ as well as the up-regulated miR-206 (involved in cardiac and skeletal muscle development)^36^. Interestingly, these miRNAs were not found to be altered in the already existing array- or sequencing-based studies based on right ventricular tissue of TOF patients^17,18,20,24^. This is surprising and might be explained by technical aspects such as platform (array versus sequencing), normalization strategy and read count statistic. In particular, the last two aspects are important for highly expressed miRNAs such as miR-1 with more than one million reads for each sample. In general, the overlap with other TOF studies, which are already very heterogeneous in respect to their altered miRNAs, is limited with 25%, and only miR-222-3p overlaps all studies (Fig. 3). The miRNA-221/222 family may target several genes involved in TGF-β signaling and recently it was shown that heart failure-associated down-regulation of this miRNA family enables profibrotic signaling in the pressure-overloaded heart^37^. Our TOF patients have been selected at a non-failing heart stage that is in line with a 3-fold up-regulation of miR-222-3p. Among technical aspects, the low overlap of the different studies could also be explained by gender, age, sample size and ethnic backgrounds^14^. More importantly, it reflects the complexity of the disease. The four abnormalities in TOF can all exhibit variable levels of severity and as a result, no two TOF cases are the same. We assigned subgroups of TOF cases based on their phenotypic features. Linear modeling techniques revealed influences of these subgroups on specific gene expression alterations^38^. Thus, we analyzed a homogenous group of TOF patients and normal heart controls in terms of their clinical parameters and features (Supplementary Fig. S1).

MiRNAs have also been described to be differently expressed and to regulate different cell types and pathways during cardiac aging^39,40^. In particular, the cardiac miR-21 is up-regulated with age in mice, and overexpression of Argonaut proteins synergistically induced miR-21. In general, major changes occurred later in life, from middle- to old-aged mice, and half of the candidate miRNAs were clustered^39,40^. A miRNA analysis on human skeletal muscle biopsies of young (~31 y) and older adults (~73 y) also revealed age-related differences (e.g., let-7 family members), and dysregulated miRNAs are related to genes associated with cell cycle, inflammation and stress^41^. For ethical reasons, it is highly difficult to have high quality cardiac samples of young children. This is further reflected by the limited availability of normal cardiac datasets from young children in general. The only solution to overcome this bottleneck is a conservative analysis and consideration of different aspects. In our study, we compared infant TOF hearts (0–3 y) to young/mid-age adult normal hearts. Our dysregulated miRNAs are not clustered (Fig. 2B). Moreover, we did not compare infant hearts to old adult hearts, where one could expect an increase of age-related pathways involved in for example cell cycle, inflammation or oxidative stress. Nevertheless, among possible age-related differentially expressed miRNAs (i.e., 5 out of 172) are miR-21 and let-7a. As mentioned above, miR-21 is up-regulated with age^39,40^ and a higher let-7 expression was described as a possible indicator of impaired cell cycle function possibly contributing to reduced muscle cell renewal and regeneration in older human muscle^41^. However, it cannot be clearly determined whether differential expression of these miRNAs between TOF and normal heart is age- or disease-related. We strongly believe that our results build a good and solid basis for follow-up studies in humans and animals as well as cell culture models, which have to verify the causative impact of these dysregulated miRNAs.

To further reduce the list of significantly expressed miRNAs and to find candidates with impact on gene expression in TOF, target prediction incorporating significantly expressed mRNAs of the same patient and control cohort was performed. Combining miRNA and mRNA expression has been shown to be efficient to identify the top candidates out of a huge number of putative targets for each miRNA^42^. In addition, only negative correlated miRNA-mRNA pairs were considered in this study as candidates, although miRNA-mRNA pairs can also be positively correlated und functional relevant^43^. Indeed, the proportion of positively and negatively correlated miRNA-mRNA pairs is equally distributed. However, the 344 negative correlated pairs were further reduced considering probabilistic miRNA–mRNA interaction signatures. Out of hundreds of expressed miRNAs and thousands of expressed mRNAs in the TOF patients or normal heart controls, only 5 miRNAs and 18 genes negatively correlated in 19 pairs might have a significant probability to be disease-relevant in the TOF patients. Among them are for example miR-1 with the main K+ channel subunit responsible for setting and maintaining the cardiac resting membrane potential (*KCNJ2*), miR-133b with a component of connective tissue microfibrils (*FBN2*; Fibrillin 2) or a sodium-dependent glutamine transporter (*SLC38A3*; Solute Carrier Family 38 Member 3), and miR-133b or miR-140-5p with the slow skeletal inhibitory subunit of the troponin complex (*TNNI1* (Troponin I1, Slow Skeletal Type). Of note, we showed that the sarcomeric gene *TNNI1* is also affected by DNA methylation changes co-localized with novel, differential splicing events in these TOF patients^5^.

**Figure 6:**
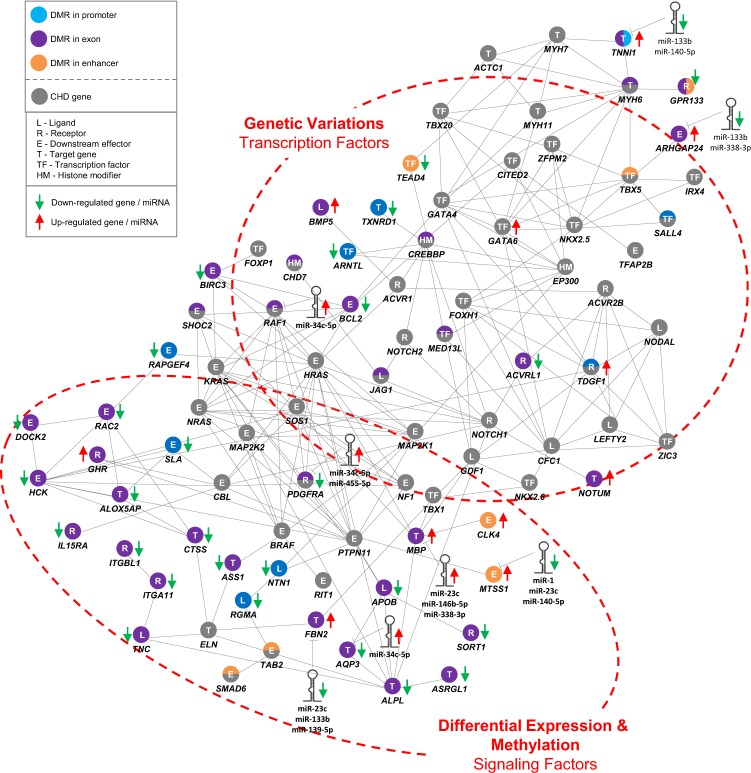
Multilevel interaction network in TOF patients extended by significantly altered miRNAs. The network is based on the study by Grunert *et al*.^5^. The known mutated genes in CHD patients comprise a high number of transcriptional regulators (transcription factors and histone modification), while the connected genes with differential expression and methylation and/or targeted by altered miRNAs in the right ventricular tissue of TOF patients compared to normal heart controls consist mainly of downstream targets and signaling molecules. DMR indicates differentially methylated region; CHD, congenital heart disease.

The overlap of differentially expressed miRNA and mRNA expression profiles could dramatically reduce the huge number of putative mRNA targets potentially disease-relevant genes. However, translational repression and target mRNA degradation by miRNAs can only be one small aspect to fully understanding a complex phenotype like TOF. MiRNAs are part of larger co-regulation and interaction networks, which are influenced by multiple factors such as genomic variations, DNA methylation, other non-coding RNAs such as piwi-interacting RNAs or even alternative splicing events as in the case of TNNI1. This is also reflected by miRNA extension to a multilevel interaction network in TOF (Fig. 6)^5^. The network comprises mutated CHD genes and significantly differentially expressed and methylated genes or miRNAs (based on the 344 miRNA-mRNA pairs) in the TOF patients. In summary, genome-wide miRNA and mRNA expression profiles of TOF patients and normal heart controls were investigated. We found several known and few novel altered miRNAs with respectively altered target genes in the right ventricle of TOF patients. Our data suggest disease-relevant miRNA-mRNA pairs that are open to further investigations in heart tissue as well as circulating in the blood.

## Methods

### Ethics statement and samples

Studies of patients were performed according to the institutional guidelines of the German Heart Institute in Berlin, with approval of the ethics committee of the Charité - Universitätsmedizin Berlin and written informed consent of patients and/or parents, kin, caretakers, or guardians on behalf of the minors involved in our study. The study conforms to the principles outlined in the Declaration of Helsinki. The homogeneous cohort of isolated TOF cases (i.e., without any additional cardiovascular or other abnormalities) was selected based on our previous evaluations^38,44^. Moreover, the number of samples derived from male and female individuals is almost equally distributed for both TOF patients and controls (Supplementary Fig. S1; 2 female and 2 male normal hearts (left and right ventricular samples each from the same heart) as well as 10 female and 12 male TOF samples). Myocardial biopsies were taken during the first corrective surgery after short-term cardioplegia. Samples were collected in collaboration with the German Heart Institute in Berlin and directly snap-frozen in liquid nitrogen after excision and stored at −80 °C. Tissue from normal human hearts was obtained from unmatched organ donors without cardiac disease, where the hearts could not be transplanted because of organizational difficulties.

### Small RNA sequencing, read mapping, annotation and quantification

The total RNA was isolated and prepared for small RNA sequencing according to manufacturer’s protocol (Illumina 11251913 Rev. A; “Preparing Samples for Analysis of Small RNA”). Sequencing libraries were generated and purified DNA was used directly for sequencing. 36 bp single-end read sequencing was performed using the Illumina’s Genome Analyzer. The sequencing and probability output files were converted to FASTQ format using MAQ^45^. The quality of the sequencing data was checked using FASTQC (http://www.bioinformatics.babraham.ac.uk/projects/fastqc/). All samples passed sequence quality.

After initial quality check, the reads were mapped to the human reference genome (hg19) using MicroRazerS^46^. The parameters were set as follows: -m 20 (maximum number of best matches), -pa (purge ambiguous reads having more than 20 equally-best hits) and -sL 18 (seed length for miRNA of length 19–25 nt). In addition, reads can have at most one error in the seed sequence to be robust towards possible sequencing errors and sequence variations. As MicroRazerS is prefix-based, meaning that the read mapper searches for the longest contiguous match starting at the first read base, no adapter trimming is required. Moreover, this mapping process is robust to possible sequencing errors, which especially occur at the 3′ end of reads^46^.

Mapped reads are annotated based on their overlap to known genomic annotations including precursor and mature miRNAs, transfer RNAs (tRNA), C/D box & H/ACA small nucleolar RNAs (snoRNAs), cajal body-specific RNAs (scaRNAs), ribosomal RNAs (rRNAs), small cytoplasmic RNAs (scRNAs), small nuclear RNAs (snRNAs), miscellaneous other RNAs (miscRNAs), mitochondrial tRNAs (Mt-tRNAs), piwi-interacting RNAs (piRNAs), mRNAs and repeats. Annotations are obtained from the miRBase v20 (GRCh37) and UCSC database (tRNA, rnaGene, wgrna, knownGene and rmsk tracks; GRCh36/37). If a read overlapped to known mature/precursor miRNAs it was assumed that the read was a sequencing product of this miRNA and the read was added to its read count. All other small RNA classes were annotated in the same manner and ordered as mentioned above. Reads which could not be overlapped with any known annotations were declared as unknown sequences.

### Differential expression analysis

To perform differential expression analysis, the miRNA read counts were normalized using the TCC method, which incorporates an overabundance of some heart-related miRNAs^47^. After removing outliers (TOF-09 and NH-03) based on multi-dimensional scaling, a negative binomial (gamma-Poisson) model was applied to miRNAs with a minimal tag count of more than 50 over all analyzed samples. An exact test was conducted to test differential expression appropriate for the negative binomially distributed miRNA read counts^48^. The observed p-values from gamma-Poisson tests of TCC normalized miRNA read counts follow a normal distribution (Supplementary Fig. S8A) and were further adjusted using the Benjamini and Hochberg (BH) correction method with a false discovery rate (FDR) smaller than 5%. Significantly differentially expressed miRNAs have a fold change above 1.5 (Supplementary Fig. S8B).

### Target prediction

To predict miRNA targets, the tools MIRZA-G^27^ and TargetScanHuman^28^ were used. Already pre-computed lists of targets genes for all human miRNAs were used for both tools and filtered for the set of significantly differentially expressed miRNAs (n = 172) and mRNAs (n = 972) in right ventricular tissue of TOF patients and normal heart controls. Note that the mRNA-seq data from the same TOF and normal hearts were already published by us and the data analysis including differential expression are described elsewhere^5,7^. As MIRZA-G combines all miRNA-mRNA pairs in one single pair, the same approach was applied to all TargetScan pairs. TargetScanHuman was adapted to report all miRNAs belonging to a miRNA family. All transcript scores for one gene were combined to one gene score. The score was taken from best score over all transcripts. The scores of MIRZA-G ranged between 0 and 3.6 and for TargetScan between −3 and −0.02. No additional thresholds were applied to the scores. The predicted miRNA-mRNA pairs were further filtered applying a probabilistic miRNA-mRNA interaction signature (ProMISE) approach^32^ using all expressed miRNAs (n = 657 with TCC > 1 in at least one individual) and mRNA (n = 11,977 with Reads Per Kilobase Million (RPKM) > 1 in at least one individual) in TOFs and/or controls. Only pairs with a ProMISE score > 0.004 were used (~90% quantile in NH-rv and ~87% quantile in TOF-rv). Over-representation analysis for GO terms and biological pathways based on genes identified in miRNA-mRNA pairs after ProMISE filtering was performed using ClueGO (adjusted p-value ≤ 0.05 using hypergeometric test with correction for multiple testing using Benjamini-Hochberg method)^49^.

### Novel miRNA prediction

The fold- and scoring-based approach of the miRDeep package was used to identify novel miRNAs^33^. Briefly, all read sequences with a mapped read length of less or equal than 25 nt (longer sequences are unlikely to represent mature miRNA sequences) as well as a sequence count of more than 25 (removing noise) which are not annotated to known miRNAs or other small non-coding RNAs were used for novel miRNA prediction. Within the miRDeep approach, each potential miRNA precursor sequence was assessed after folding into a hairpin structure using the RNA folding algorithm from the ViennaRNA package^50^. Furthermore, miRDeep searches for potential cleavage sites of Drosha and Dicer, and uses phylogenetic conservation and a filtering of other known small non-coding RNA species to improve the predictions. The stability of potential precursors sequences is tested using Randfold v2.0^51^. In summary, each potential miRNA precursor sequence was scored based on its read signature, secondary structure (e.g., multi-loops, minimum free energy, etc.), cleavage, conservation and overlap to known small non-coding RNAs. The novel miRNA candidates were further subjected to differential expression analysis along all known miRNAs (TCC normalized counts, negative binomial testing, multiple testing, adjusted p-value < 0.05). For each novel miRNA, target prediction was performed based on TargetScanHuman^28^ using differential expressed mRNAs in TOF-rv versus NH-rv and filtering by an aggregated PCT score > 0.8, a context++ score ≤0.4 and a percentile >85^52^. For the sets of target genes, on over-representation analysis for GO terms and pathways was performed using ClueGO^49^ and ConsensusPathDB^53^ (adjusted p-value ≤ 0.05 using hypergeometric test with correction for multiple testing using Benjamini-Hochberg method).

## Supplementary information

Supplementary information

## Data Availability

Small RNA-seq data has been submitted to the Gene Expression Omnibus (GEO) repository at NCBI (accession number GSE36759). RNA-seq data for TOF-rv and NH-rv samples are also available at the GEO repository (accession number GSE36761).
