# Supplementary material for: Altered microRNA and target gene expression related to Tetralogy of Fallot
Source: Sci Rep. 2019 Dec 13;9:19063. doi: 10.1038/s41598-019-55570-4 (PMC6911057; doi:10.1038/s41598-019-55570-4)
Supplement: Supplementary file 1 — Supplementary information [file 41598_2019_55570_MOESM1_ESM.pdf]

Altered microRNA and target gene expression related to Tetralogy of Fallot

Marcel Grunert, Sandra Appelt, Ilona Dunkel, Felix Berger & Silke R. Sperling

Supplementary Information

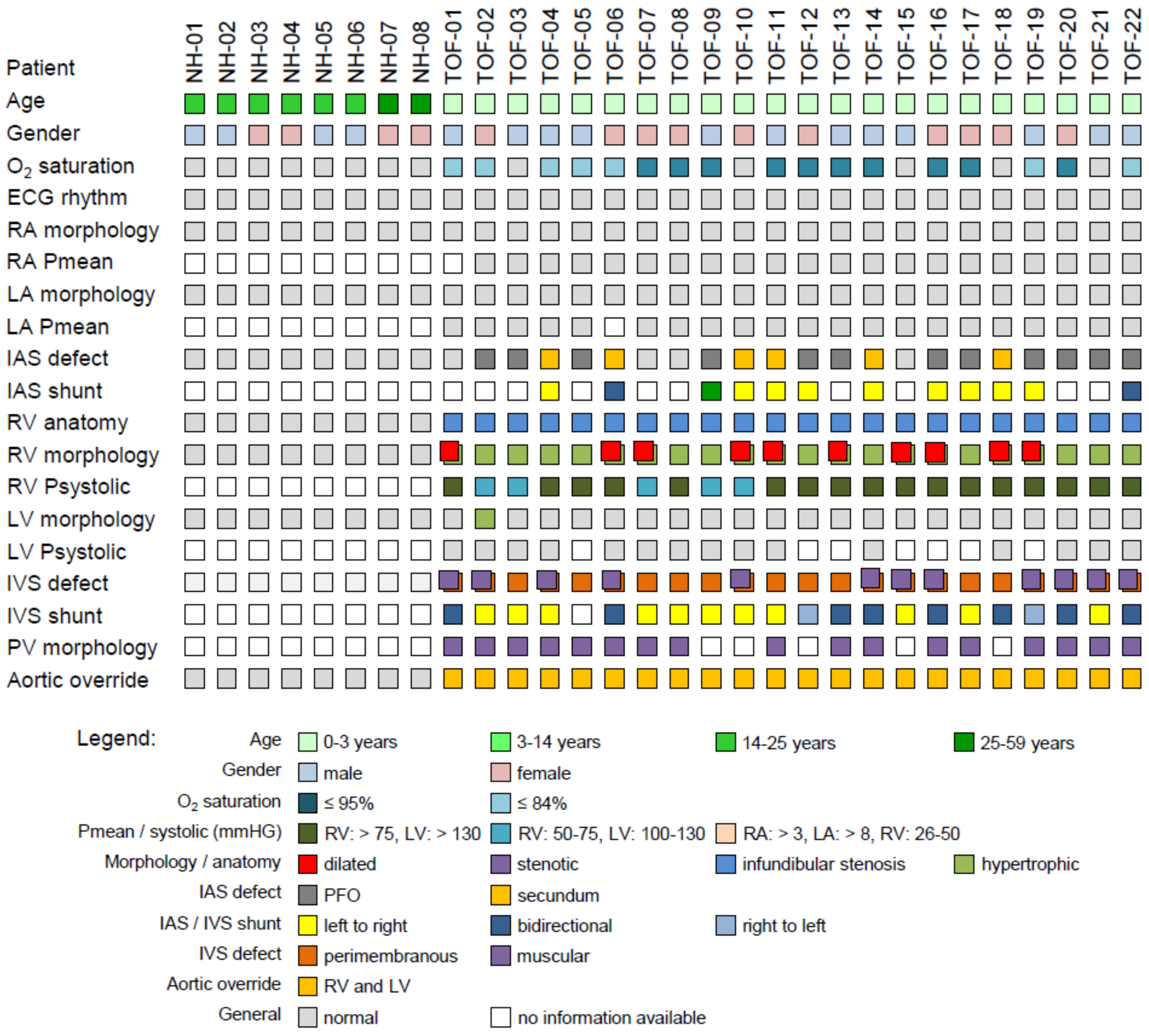

Supplementary Figure S1. Phenotype matrix for TOF patients and normal heart controls. Each individual is represented by one column. In addition to general information, all pathological features are indicated. Double boxes are used for more than one piece of information per row. See legend for color-coding. The samples NH-01, NH-03, NH-05 and NH-07 were taken from left ventricle whereas all other normal heart and TOF samples were taken from right ventricle. ECG indicates electrocardiogram; IAS, intra-atrial septum; IVS, intra-ventricular septum; LA, left atrium; LV, left ventricle; NH, normal heart; PFO, patent foramen ovale; Pmean, mean pressure; Psystolic, systolic pressure; PV, pulmonary valve; RA, right atrium; RV, right ventricle; TOF, Tetralogy of Fallot.

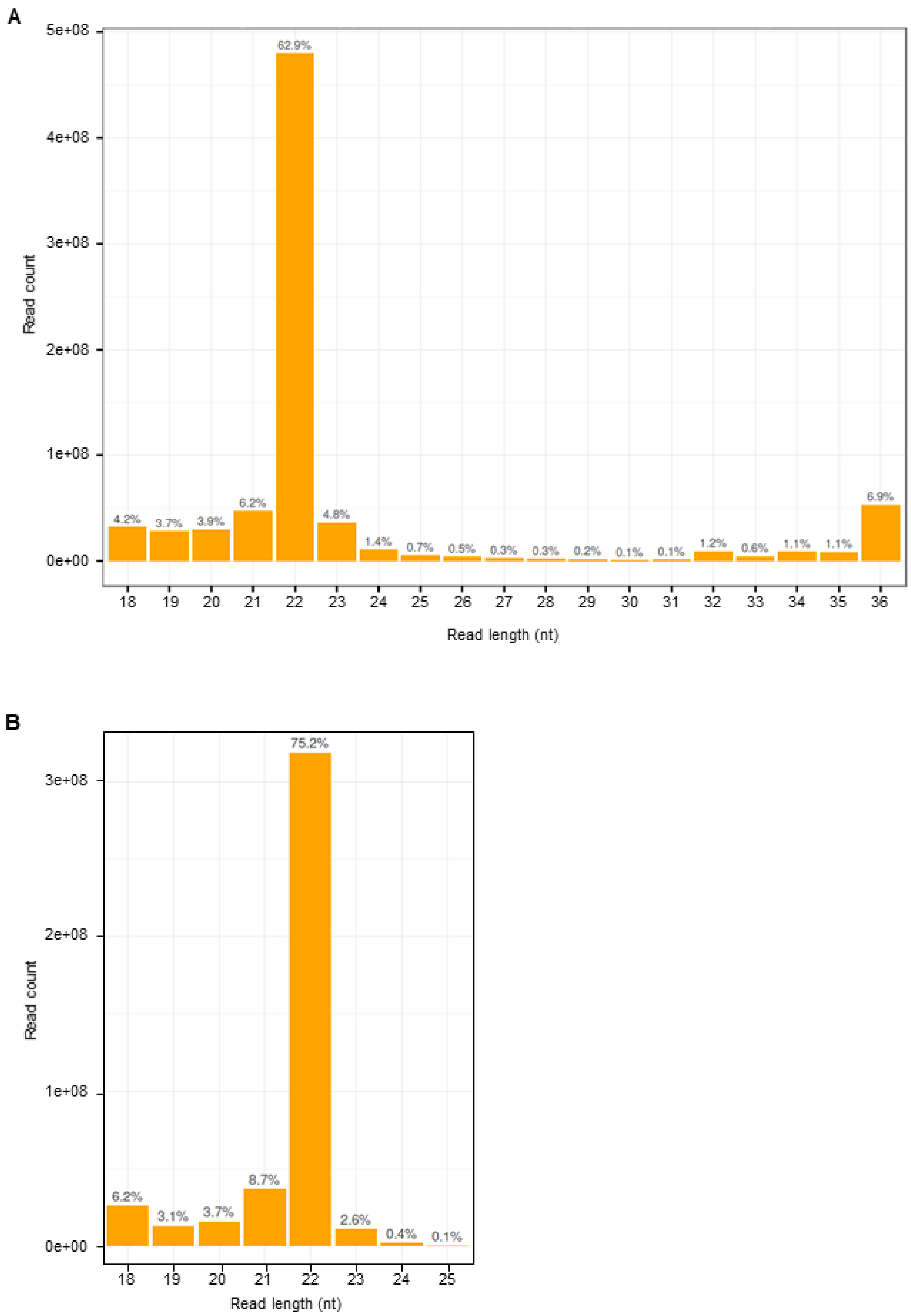

Supplementary Figure S2. Read length distribution over all TOF-rv, NH-lv and NH-rv samples of (A) all mapped sequences and (B) mapped mature miRNA sequences. nt: nucleotides.

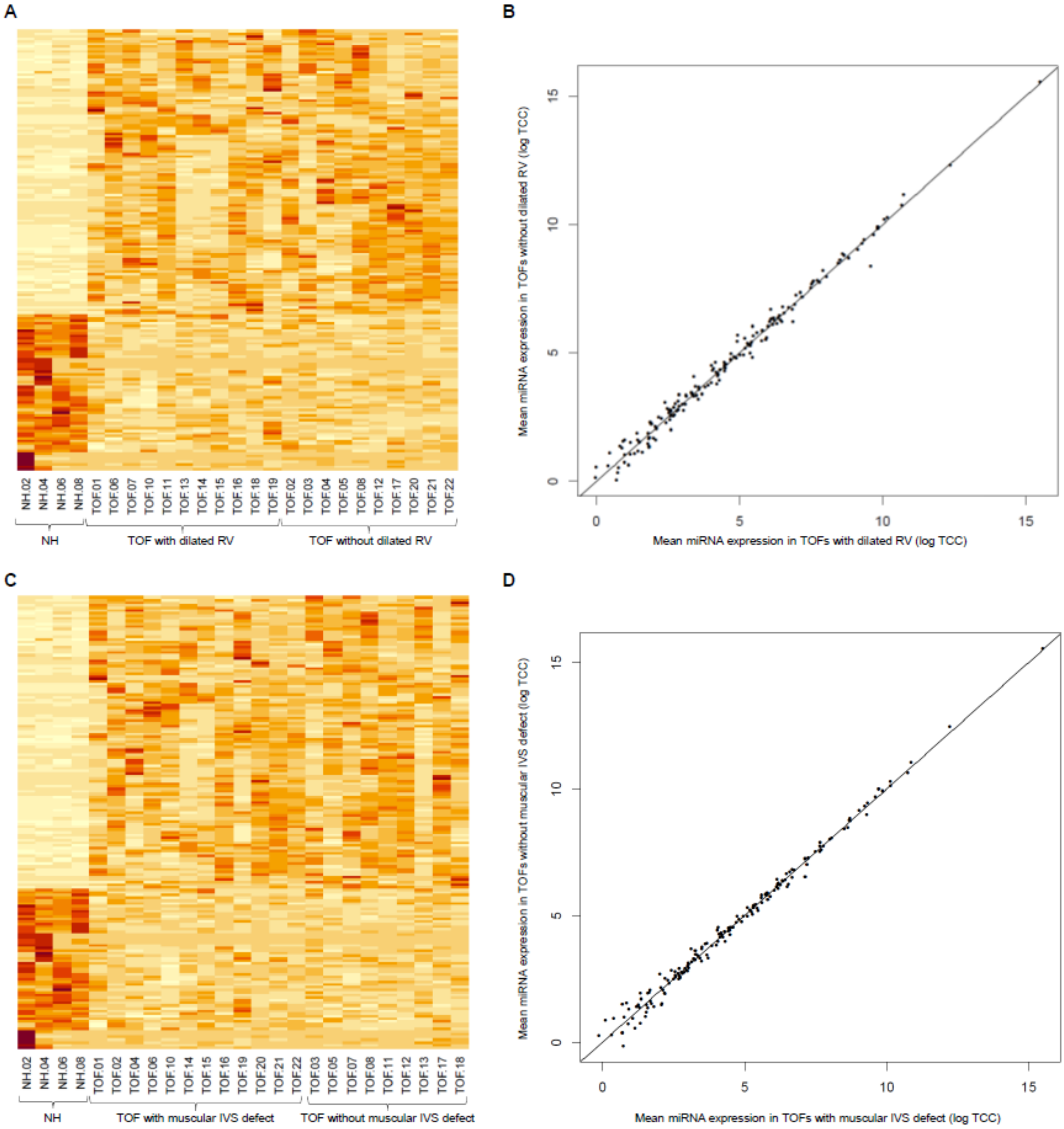

Supplementary Fig. S3: Expression of significantly differentially expressed miRNAs (n=172; TOF-rv vs. NH-rv) in TOFs with and without dilated RV or muscular IVS defect. (A+C): Heatmap based on hierarchical clustering of identified miRNAs (TCC normalized expression values) among the different groups of samples/individuals. (B+D): MiRNA correlation of expression values in the two groups of patients. IVS: intra-ventricular septum; NH: normal heart (samples from right ventricle); RV: right ventricle; TOF: Tetralogy of Fallot (samples from right ventricle).

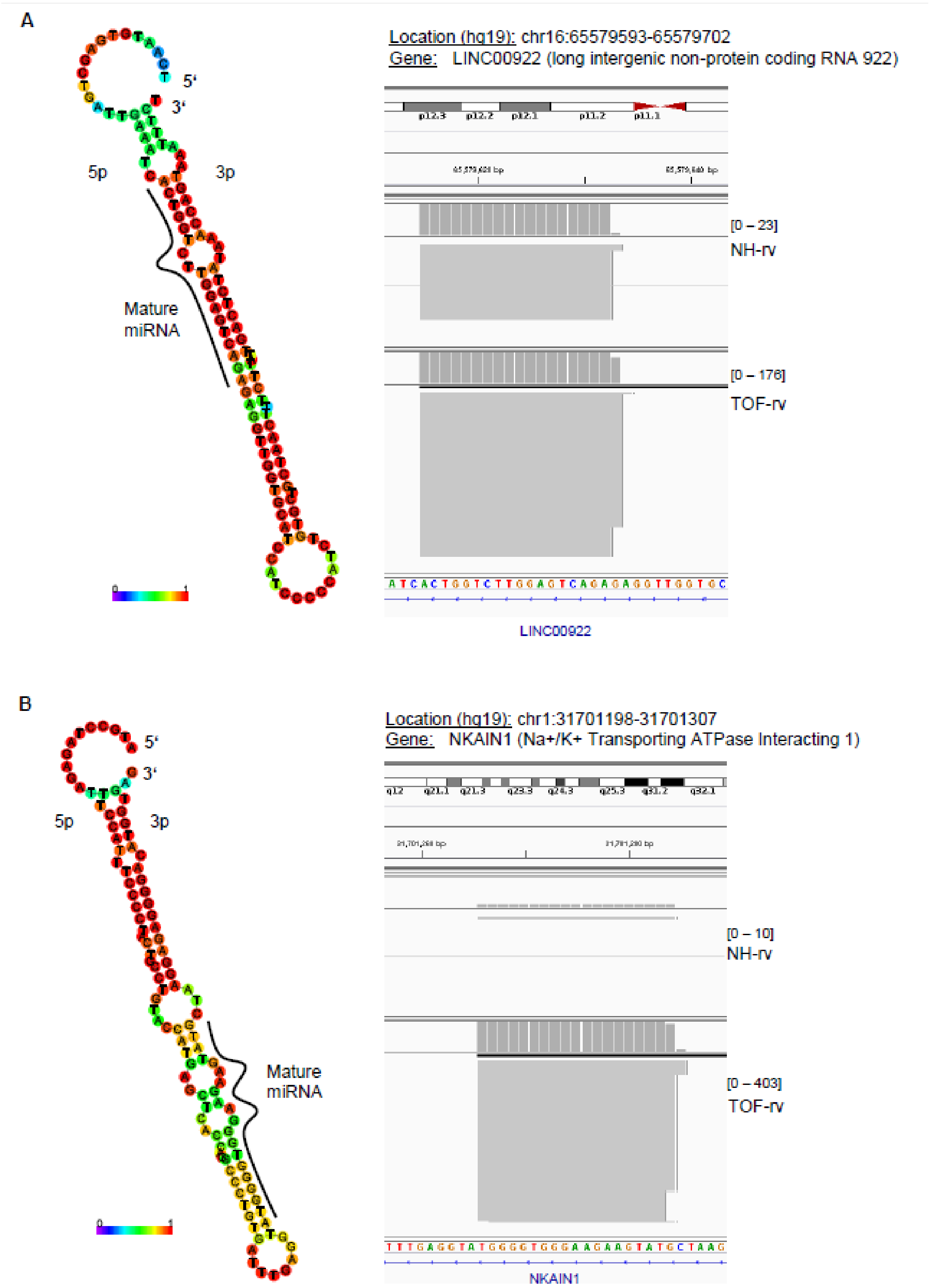

Supplementary Figure S4. Predicted novel miRNA candidates. (A) Novel miRNA located at chromosome 16 and overlapping the long non-coding RNA LINC00922. (B) Novel miRNA located at chromosome 1 and in an intron of NKAIN1. (Left) Centroid secondary structure drawing encoding base-pair probabilities based on RNAfold WebServer. (Right) Graphical representation of the novel miRNA location with read count over all NH-rv and TOF-rv samples.

#miRDeep information for novel miRNA located at chr6:39074296-39074405

score_nucleus 3
score_star -1.3
score_mfe 1.9
score_freq 0
score 3.9
flank_first_end 17
flank_first_seq TGTGCCTGGAGCCCAGC
flank_first_struct ...((..(((((.((((
flank_second_beg 88
flank_second_seq TGTGCTTCTGCCTCTCACCACGT
flank_second_struct)).))))).))............
freq 207
loop_beg 41
loop_end 64
loop_seq TTTGCCAAGAACCATTGTGTCTTT
loop_struct ...(((((......))).))..))
mature_arm second
mature_beg 65
mature_end 87
mature_query read_18809047_x94
mature_seq TTTTTTGCTGGAACATTTCTGGT
mature_strand +
mature_struct))))).)))))))))))))..))
pre_seq TGGAAATGTTCTAGCCAAAAAAGTTTGCCAAGAACCATTGTGTCTTTTTTTTTGCTGGAACATTTCTGGT
pre_struct ..(((((((((((((.(((((((...(((((......))).))..))))))).)))))))))))))..))
pri_beg 1
pri_end 110
pri_id chr6_705
pri_mfe -43.30
pri_seq
TGTGCCTGGAGCCCAGCTGGAAATGTTCTAGCCAAAAAAGTTTGCCAAGAACCATTGTGTCTTTTTTTTTGCTGGAACATTTC
TGGTTGTGCTTCTGCCTCTCACCACGT
pri_struct ...((..(((((.((((..(((((((((((((.(((((((...(((((......))).))..))))))).)))))))))))))..)).))))).))............
star_arm first
star_beg 18
star_end 40
star_seq TGGAAATGTTCTAGCCAAAAAAG
star_struct ..(((((((((((((.(((((((

Supplementary Figure S5. Predicted novel miRNA candidate located at chromosome 6 and in an intron of GLP1R. Given are the various miRDeep information.

#miRDeep information for novel miRNA located at chr16:65545690-65545799

score_nucleus -0.6
score_star -1.3
score_randfold 1.6
score_mfe 1.1
score_freq 0
score 1.1
flank_first_end 22
flank_first_seq TCAATGTGAGCTGATTGAAATC
flank_first_struct ................(((((.
flank_second_beg 105
flank_second_seq ATTTCT
flank_second_struct))))).
freq 50265
loop_beg 41
loop_end 85
loop_seq GAGGTTGGTGCATCCATCCCCCATCTGTGCTGCTAACTTTCTTAT
loop_struct (((((((((((((.............)))).)))))).)))))..
mature_arm first
mature_beg 23
mature_end 40
mature_query read_41555275_x12339
mature_seq ACTGGTCTTGGAGTCAGA
mature_strand +
mature_struct ((((((..((((((((((
pre_seq
ACTGGTCTTGGAGTCAGAGAGGTTGGTGCATCCATCCCCCATCTGTGCTGCTAACTTTCTTATTGACTCTATAAACCAGTAA
pre_struct ((((((..(((((((((((((((((((((((.............)))).)))))).)))))..))))))))...))))))..
pri_beg 1
pri_end 110
pri_id chr16_1130
pri_mfe -33.22
pri_seq
TCAATGTGAGCTGATTGAAATCACTGGTCTTGGAGTCAGAGAGGTTGGTGCATCCATCCCCCATCTGTGCTGCTAACTTTCTT
ATTGACTCTATAAACCAGTAAATTTCT
pri_struct ................(((((.((((((..(((((((((((((((((((((((.............)))).)))))).)))))..))))))))...))))))..))))).
star_arm second
star_beg 86
star_end 104
star_seq TGACTCTATAAACCAGTAA
star_struct))))))))...))))))..

Supplementary Figure S6. Predicted novel miRNA located at chromosome 16 and overlapping the long non-coding RNA LINC00922. Given are the various miRDeep information.

```
#miRDeep information for novel miRNA located at chr1:31228351-31228460

score_nucleus	3
score_star	-1.3
score_mfe	1.4
score_freq	0
score	3.4
flank_first_end	38
flank_first_seq	ATGCCTAGAGATTTCCATTTCCCCTACTGCCTGTACCA
flank_first_struct	...........((.((((.((((((.((.(((....((
flank_second_beg	88
flank_second_seq	CTAAGGAGAGGGGACATGGTGAG
flank_second_struct	...))))))))))).)))).)).
freq	388
loop_beg	59
loop_end	68
loop_seq	ATTTGAGGTA
loop_struct	........))
mature_arm	second
mature_beg	69
mature_end	87
mature_query	read_65782186_x255
mature_seq	TGGGGTGGGAAGAAGTATG
mature_strand	+
mature_struct	))))))))..))...))))
pre_seq	TGAGCTCACCAGGCCCTGTGATTTGAGGTATGGGGTGGGAAGAAGTATG
pre_struct	((..((..((..((((((((........))))))))))..))...))))
pri_beg	1
pri_end	110
pri_id	chr1_542
pri_mfe	-36.20
pri_seq
	ATGCCTAGAGATTTCCATTTCCCCTACTGCCTGTACCATGAGCTCACCAGGCCCTGTGATTTGAGGTATGGGGTGGGAAGAA
GTATGCTAAGGAGAGGGGACATGGTGAG
pri_struct	...........((.((((.((((((.((.(((....((((..((..((..((((((((........))))))))))..))...))))...))))))))))).)))).)).
star_arm	first
star_beg	39
star_end	58
star_seq	TGAGCTCACCAGGCCCTGTG
star_struct	((..((..((..((((((((
```

Supplementary Figure S7. Predicted novel miRNA candidate located at chromosome 1 and in an intron of NKAIN1. Given are the various miRDeep information.

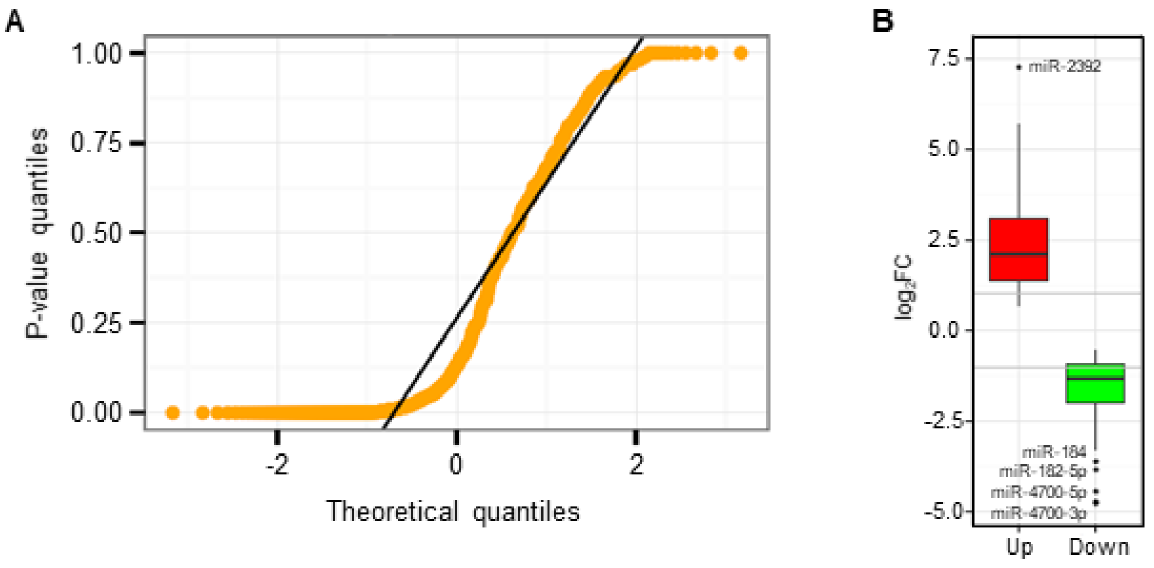

Supplementary Figure S8. Distribution of p-values and fold changes for significantly differentially expressed miRNAs in TOF-rv versus NH-rv. (A) QQ-plot for p-values derived from exact test for negative binomially distributed read counts after TCC normalization. (B) Fold change (FC) of up- (red) and down-regulated (green) miRNAs.

miRNA	q-value	m-value	FC
hsa-let-7a-3p	3.04E-02	-0.82	-1.77
hsa-miR-1	7.16E-03	-0.68	-1.61
hsa-miR-10a-5p	2.99E-13	-2.89	-7.40
hsa-miR-124-3p	4.54E-05	-2.96	-7.79
hsa-miR-126-3p	9.06E-03	-0.77	-1.70
hsa-miR-133b	6.88E-04	-1.51	-2.84
hsa-miR-135a-5p	3.98E-02	-1.26	-2.39
hsa-miR-139-3p	7.72E-04	-1.47	-2.77
hsa-miR-139-5p	1.64E-09	-1.78	-3.44
hsa-miR-140-3p	3.33E-02	-0.54	-1.45
hsa-miR-140-5p	3.86E-10	-2.01	-4.02
hsa-miR-143-5p	8.61E-03	-0.64	-1.56
hsa-miR-146b-5p	6.97E-04	-1.32	-2.50
hsa-miR-148a-3p	1.39E-10	-1.57	-2.97
hsa-miR-148b-3p	1.65E-11	-1.65	-3.14
hsa-miR-150-5p	2.02E-03	-1.12	-2.17
hsa-miR-151a-5p	3.36E-02	-0.58	-1.50
hsa-miR-151b	4.54E-05	-1.20	-2.30
hsa-miR-182-5p	3.92E-09	-4.45	-21.79
hsa-miR-183-5p	8.42E-06	-3.62	-12.27
hsa-miR-184	3.72E-07	-3.85	-14.38
hsa-miR-187-5p	4.18E-03	-2.39	-5.24
hsa-miR-193b-3p	5.24E-03	-0.89	-1.86
hsa-miR-215-5p	1.71E-02	-1.34	-2.53
hsa-miR-21-5p	6.82E-03	-0.76	-1.69
hsa-miR-223-3p	4.61E-05	-1.14	-2.20
hsa-miR-22-3p	1.06E-04	-0.89	-1.86
hsa-miR-23c	1.33E-08	-2.72	-6.58
hsa-miR-29a-3p	7.46E-04	-1.00	-2.01
hsa-miR-29b-3p	3.16E-16	-2.32	-4.99
hsa-miR-29c-3p	5.22E-05	-1.35	-2.55
hsa-miR-29c-5p	1.87E-03	-1.10	-2.14
hsa-miR-3065-5p	2.26E-04	-1.10	-2.14
hsa-miR-30a-5p	2.54E-02	-0.71	-1.64
hsa-miR-30c-2-3p	8.26E-04	-0.82	-1.77
hsa-miR-30d-5p	1.67E-02	-0.83	-1.78
hsa-miR-320d	3.45E-02	-1.03	-2.05
hsa-miR-338-3p	2.26E-04	-1.10	-2.14
hsa-miR-345-3p	1.25E-05	-3.27	-9.65
hsa-miR-34a-3p	1.20E-02	-1.63	-3.09
hsa-miR-3690	8.84E-03	-1.63	-3.10
hsa-miR-4485	2.83E-03	-1.54	-2.90
hsa-miR-4521	9.77E-04	-1.49	-2.81
hsa-miR-4532	2.85E-08	-2.34	-5.07
hsa-miR-4634	3.08E-02	-1.22	-2.33
hsa-miR-4700-3p	2.32E-10	-4.78	-27.39
hsa-miR-4700-5p	1.65E-11	-4.72	-26.44
hsa-miR-598-3p	7.32E-03	-0.82	-1.76
hsa-miR-628-5p	1.76E-02	-1.12	-2.17
hsa-miR-642a-3p	4.51E-02	-0.94	-1.91
hsa-miR-642b-5p	4.56E-02	-0.94	-1.91
hsa-miR-6503-5p	3.66E-03	-1.45	-2.74
hsa-miR-6723-5p	7.72E-04	-1.47	-2.77
hsa-miR-676-5p	3.83E-02	-1.07	-2.10
hsa-miR-7704	3.39E-02	-0.76	-1.69
hsa-miR-7975	2.45E-03	-2.29	-4.88
hsa-miR-8062	1.90E-04	-2.33	-5.01
hsa-miR-9-3p	5.63E-03	-1.31	-2.48
hsa-miR-940	2.02E-03	-1.44	-2.72
hsa-miR-98-3p	1.22E-03	-1.58	-3.00
hsa-miR-98-5p	8.78E-03	-2.16	-4.47

miRNA	q-value	m-value	FC
hsa-let-7i-5p	8.18E-10	2.23	4.68
hsa-miR-101-5p	4.00E-02	1.46	2.75
hsa-miR-105-5p	3.16E-02	1.67	3.19
hsa-miR-122-3p	1.56E-03	1.89	3.71
hsa-miR-1261	3.27E-08	4.19	18.29
hsa-miR-1262	3.17E-03	1.43	2.70
hsa-miR-126-5p	1.01E-03	1.39	2.63
hsa-miR-1271-3p	8.42E-06	3.53	11.53
hsa-miR-127-3p	3.33E-02	0.85	1.81
hsa-miR-1285-3p	3.41E-02	1.18	2.27
hsa-miR-130a-3p	1.97E-10	1.69	3.23
hsa-miR-130b-3p	4.23E-03	1.14	2.20
hsa-miR-136-5p	5.77E-05	4.52	22.92
hsa-miR-144-3p	1.26E-03	2.54	5.83
hsa-miR-146a-5p	4.00E-02	1.29	2.44
hsa-miR-15b-5p	1.81E-02	1.26	2.39
hsa-miR-181a-5p	2.83E-03	0.82	1.76
hsa-miR-181b-5p	2.23E-08	1.76	3.39
hsa-miR-181c-3p	1.20E-02	0.90	1.87
hsa-miR-181c-5p	2.32E-02	0.94	1.91
hsa-miR-181d-5p	9.91E-11	2.26	4.80
hsa-miR-1827	9.58E-04	3.41	10.61
hsa-miR-186-5p	1.53E-02	0.89	1.85
hsa-miR-187-3p	2.10E-05	3.74	13.34
hsa-miR-204-3p	6.56E-03	2.47	5.55
hsa-miR-204-5p	3.19E-05	3.05	8.31
hsa-miR-206	1.15E-05	2.85	7.23
hsa-miR-20b-3p	7.22E-04	2.63	6.17
hsa-miR-20b-5p	1.11E-04	2.62	6.14
hsa-miR-210-3p	3.46E-03	0.92	1.89
hsa-miR-221-3p	8.42E-06	2.18	4.53
hsa-miR-221-5p	1.38E-05	1.91	3.75
hsa-miR-222-3p	2.05E-06	1.50	2.83
hsa-miR-2355-3p	2.39E-03	4.00	15.98
hsa-miR-2392	1.33E-06	7.27	153.97
hsa-miR-23b-5p	2.49E-02	0.88	1.84
hsa-miR-28-3p	7.20E-07	1.71	3.26
hsa-miR-29a-5p	4.00E-02	1.59	3.02
hsa-miR-30a-3p	7.16E-03	0.90	1.87
hsa-miR-3117-3p	3.20E-07	2.56	5.90
hsa-miR-3124-5p	3.18E-02	1.12	2.18
hsa-miR-3127-5p	7.29E-04	2.04	4.11
hsa-miR-3168	4.51E-07	4.46	22.07
hsa-miR-3184-5p	3.86E-03	0.99	1.99
hsa-miR-32-5p	4.25E-07	2.20	4.59
hsa-miR-335-3p	4.72E-02	2.59	6.01
hsa-miR-33a-5p	1.54E-05	1.65	3.14
hsa-miR-33b-5p	1.18E-08	3.21	9.26
hsa-miR-34c-5p	4.60E-03	1.23	2.35
hsa-miR-3591-5p	1.55E-03	1.89	3.71
hsa-miR-3613-5p	2.37E-02	3.01	8.07
hsa-miR-3615	1.13E-02	1.39	2.61
hsa-miR-363-3p	2.10E-05	1.45	2.74
hsa-miR-371a-5p	8.42E-06	2.37	5.18
hsa-miR-371b-3p	8.42E-06	2.37	5.18
hsa-miR-372-3p	2.90E-08	3.30	9.82
hsa-miR-378a-3p	1.98E-07	2.03	4.09
hsa-miR-378b	9.56E-11	4.80	27.87
hsa-miR-378c	3.86E-10	2.12	4.33
hsa-miR-378e	1.64E-05	2.40	5.27
hsa-miR-378f	5.97E-04	2.50	5.64

miRNA	q-value	m-value	FC
hsa-miR-378g	1.62E-04	1.46	2.75
hsa-miR-378i	2.85E-05	4.25	19.02
hsa-miR-378j	2.54E-03	2.50	5.65
hsa-miR-381-3p	8.23E-05	2.10	4.28
hsa-miR-382-5p	9.56E-04	1.36	2.57
hsa-miR-3917	4.00E-02	2.72	6.59
hsa-miR-422a	3.78E-04	2.10	4.27
hsa-miR-423-3p	3.86E-03	0.99	1.99
hsa-miR-424-3p	1.71E-02	0.82	1.76
hsa-miR-424-5p	2.88E-05	2.29	4.87
hsa-miR-4306	4.81E-02	3.32	9.95
hsa-miR-433-3p	2.31E-02	0.93	1.90
hsa-miR-4429	1.90E-07	3.39	10.46
hsa-miR-4454	1.87E-02	1.74	3.33
hsa-miR-4510	6.58E-07	3.20	9.20
hsa-miR-451a	1.25E-03	2.33	5.04
hsa-miR-454-3p	1.37E-03	2.49	5.61
hsa-miR-455-5p	1.28E-04	1.36	2.57
hsa-miR-487b-5p	3.46E-03	4.07	16.78
hsa-miR-488-5p	9.93E-03	1.77	3.41
hsa-miR-493-5p	4.44E-02	1.32	2.49
hsa-miR-4999-5p	6.54E-03	3.32	9.99
hsa-miR-499a-3p	3.39E-02	0.86	1.81
hsa-miR-499b-5p	3.39E-02	0.86	1.81
hsa-miR-508-3p	3.33E-02	3.15	8.88
hsa-miR-508-5p	1.62E-03	3.60	12.10
hsa-miR-509-3-5p	1.09E-04	4.12	17.43
hsa-miR-509-3p	3.28E-02	3.01	8.05
hsa-miR-509-5p	4.03E-02	3.18	9.03
hsa-miR-542-3p	2.49E-04	1.84	3.58
hsa-miR-551b-3p	8.61E-04	1.93	3.80
hsa-miR-584-5p	6.58E-07	2.83	7.13
hsa-miR-6128	1.76E-02	1.17	2.26
hsa-miR-6129	2.59E-08	5.63	49.46
hsa-miR-6130	6.96E-06	3.09	8.50
hsa-miR-6131	1.62E-04	3.77	13.61
hsa-miR-6133	3.10E-04	5.40	42.33
hsa-miR-6134	4.76E-08	5.68	51.26
hsa-miR-618	4.46E-02	1.97	3.91
hsa-miR-6515-5p	1.00E-02	2.98	7.90
hsa-miR-663a	1.36E-02	4.69	25.72
hsa-miR-6809-3p	3.16E-02	1.28	2.43
hsa-miR-769-5p	2.92E-02	1.74	3.35
hsa-miR-8052	1.02E-02	4.07	16.74
hsa-miR-92a-3p	3.86E-03	1.04	2.06
hsa-miR-95-3p	8.42E-06	1.86	3.63
hsa-miR-95-5p	1.01E-03	3.59	12.05
hsa-miR-9-5p	1.40E-03	1.45	2.74
hsa-miR-99a-3p	3.48E-02	0.70	1.62
hsa-miR-99b-3p	6.56E-03	0.98	1.98

Supplementary Table S1. Significantly differentially expressed miRNAs in TOF-rv versus NH-rv. (Left) Down-regulated miRNAs and (center & right) up-regulated miRNAs in TOF compared to controls. MiRNAs are ordered by names. FC indicates the fold change between TOF-rv versus NH-rv.

miRNAs at chr6	miRNA at chr16	miRNA at chr1
ELK4	CAMK2B	CDC42BPG
EYS	CBFB	CHRM1
GRIN2A	HEBP1	DNAJC8
H2AFV	IL22	EEF1A1
IGF1	NBR1	EIF5A
MAF	PLEC	EXTL3
MAPK1	PTBP1	FAIM3
PLXNA4	SRA1	GTDC1
POFUT1	TLL1	HDGF
PRLR	ZFYVE26	KCND1
RBMS3		LRP4
SAMD12		MLEC
SGCD		NR4A1
VPS53		NRIP2
ZXDC		NXF1
		PALLD
		PCDHGA7
		PHB
		PLXNB2
		RNF121
		RTN4R
		SEMA4G
		SRCIN1
		SSH2
		TMEM61
		TPM3
		VAMP5
		VAT1
		YBX2
		YTHDF2
		ZCCHC17

Supplementary Table S2. Predicted target genes of the novel miRNA candidates at chromosome 6 (left, n=15), at chromosome 16 (center; n=10) and at chromosome 1 (right; n=31).
